# Guild of St. Luke's

**Published:** 1904-10-29

**Authors:** 


### GUILD OF ST. LUKE'S.

### ANNUAL MEDICAL SERVICE AT ST. PAUL'S CATHEDRAL.

The annual medical service, organised by the Guild of St. Luke, was held last Monday evening at St. Paul's Cathedral. At 7 P.M. guests were admitted by the North and South doors, and by 7.30 a large congregation had assembled. The doctors, who numbered over 120 men and a few ladies, robed at the west end of the cathedral, where they were met by the choir. During the procession the hymn "Christ is made the sure foundation" was sung. There was a large choir, but only a small number of clergy. The Chapter was represented by Archdeacon Sinclair. The service was intoned by Minor Canon J. S. Childs Clarke; the special psalm was the 91st, and the lessons were read by the Reverend T. W. Belcher, M.D., and Dr. Lewis Lewis. The preacher was the Bishop of Southwark, who took his text from Ephesians iv. 12: "For the edifying of the body of Christ." The Bishop exhorted that such a service in the cathedral of the metropolis of the greatest empire of the world was evidence that the priest of medicine and the priest of religion were ready to stand shoulder to shoulder against the forces of evil.

The object of the Guild of St. Luke is a Christian bond of union amongst Church of England members of the medical profession and promoters of medical missions; and the offertory at the service was taken in aid of the mission college maintained by the Guild.

